# Clinical trial for conventional medicine integrated with traditional Chinese medicine (TCM) in the treatment of patients with chronic kidney disease

**DOI:** 10.1097/MD.0000000000020234

**Published:** 2020-05-22

**Authors:** Yi Xi, Xun Lu, Like Zhu, Xiaoyi Sun, Yuqin Jiang, Weiming He, Minggang Wei

**Affiliations:** a The First Affiliated Hospital of Soochow University; b Suzhou Municipal Hospital, Suzhou; c Zhangjiagang TCM Hospital, Zhangjiagang; d Affiliated Hospital of Nanjing University of Chinese Medicine, Nanjing, China.

**Keywords:** Chinese herbs mixture, chronic kidney disease (CKD), Qi Gui Yi Shen decoction (QGYS), randomized clinical trial

## Abstract

**Background:**

The prevalence of chronic kidney disease (CKD) has been rapidly increasing and has become one of the most concerned global health problems. It is of good importance to improve therapeutic efficiency of CKD and delay disease progression to end stage renal disease (ESRD). Traditional Chinese Medicine (TCM) is a widely used complementary therapy for patients with CKD. The aim of this study is to evaluate whether basic treatment combined with Chinese herbs mixture Qi Gui Yi Shen decoction could achieve better therapeutic effect on CKD patients.

**Methods:**

To determine whether traditional Chinese medicine Qi Gui Yi Shen decoction could achieve better therapeutic effect, we will conduct a randomized controlled trial. A total of 100 CKD patients that meet the inclusion criteria will be enrolled and divided into 2 groups: Qi Gui Yi Shen group (QGYS group) and placebo group. Each group will receive 6-monthly basic treatment in combination with TCM or placebo 3 times per day. Efficacy of Qi Gui Yi Shen decoction is evaluated by analyzing renal function and TCM symptoms, other efficacy assessments include serum level of PAI-I, expression of transforming growth factor beta1 (TGF-beta1). Routine blood count, plasma albumin (ALB), and alanine transaminase (ALT) are evaluated as side effect and safety profile.

**Discussion:**

The results from the clinical trial will provide evidence for the effectiveness and safety of Qi Gui Yi Shen Decoction as a treatment for CKD patients. Furthermore, this will propose a new theory and method for CKD treatment.

**Trial registration:**

Registered with Chinese Clinical Trials Registry at www.chictr.org. (Registration number: ChiCTR1900021622) on 1 March 2019.

## 1 Introduction

Chronic kidney disease (CKD), also called chronic kidney failure, is described as a sustained reduction in glomerular filtration rate or evidence of structural or functional kidney abnormalities.^[1]^ According to the current international guidelines, CKD is defined as glomerular filtration rate (GFR) <60 ml/min per 1.73 m^2^ or markers of kidney injuries or both, of at least 3 months duration.^[2]^ CKD is associated with a significantly increased risk of hospital admission, morbidity, and mortality due to cardiovascular disease,^[3]^ and other complications including kidney-disease progression, acute kidney injury, cognitive decline, anemia, mineral and bone disorders, and fractures.^[4]^ Treat or not, a certain number of CKD patients would still progress to the most severe form: end stage renal disease (ESRD). The only therapeutic option for ESRD is renal replacement treatment (RRT): dialysis or transplantation. However, the mortality of patients on dialysis can be as high as 20% per year and transplantation is limited by organ shortage.^[5]^ With people's lifestyle changing, the common risks of CKD nowadays are mainly diabetes, hypertension, old age, and cardiovascular disease.^[6]^ However, no matter what causes CKD, renal fibrosis is the final common consequence and can be hardly reversed.^[7]^ Renal fibrosis now is believed to be a wound healing response to tissue damage and is usually characterized by glomerulosclerosis and tubulointerstitial fibrosis.^[8]^ Glomerulosclerosis manifests as vascular damages and loss of capillaries which will accelerate renal epithelial cells injury for nutrient intake limitation from vessels and finally leads to cell death, epithelial-to-mesenchymal cell transition and inflammatory cytokines release from these cells.^[9,10]^ Besides, overwhelming inflammatory response, in turn, leads to glomerulus impairment. Interstitial inflammation and renal tubular injury also give rise to excessive extracellular matrix (ECM) accumulation and collagen production, thus finally leads to fibrosis generation.^[11,12]^

In recent years, the prevalence of CKD has been rapidly increasing and has become one of the most concerned global health problems. In high-income countries the prevalence of CKD is about 11%. According to the epidemiologic investigation by Peking University Institute of Nephrology, the prevalence rate of CKD by 2012 in China was 10.8%.^[13]^ Yet by 2016, the total population of CKD published by National Bureau of Statistic in China reached nearly 147 million.^[14]^ However, the therapeutic options for CKD are very limited, such as protein, salt and lipid intake restriction, blood pressure control and angiotensin II receptor blockers or angiotensin converting enzyme inhibitors administration, and the efficacy is not satisfactory though. Therefore, it is of great importance to find effective and safe approaches to prevent or interfere disease progression from an early stage. Literature have suggested that for a long time Traditional Chinese Medicine (TCM) could postpone CKD progress while alleviate drug toxicity and side effects, thus improving patients’ survival and life quality.^[15]^

With the emerging worldwide interest in Chinese herbs, researchers conduct in vitro and vivo studies which have confirmed the biological activity and therapeutic effects of several Chinese herbs.^[16]^ Astragalus membranaceus (Huangqi in Chinese), for instance, is reported to be able to reduce proteinuria both in clinical and lab studies,^[17]^ and could also reduce serum cholesterol, improve immune function by regulating T cells and extenuate renal fibrosis.^[18]^ Angelica sinensis (Danggui in Chinese) is another widely studied Chinese herb, it has been shown with the ability of attenuating kidney impairment by suppressing oxidative stress and inflammation.^[19]^ Chinese herbs used for kidney diseases treatment is usually prescribed following different principles such as clearing heat and eliminating dampness and coordinating Yin and Yang in the body and the prescription varies by people of different sexes, ages, natural stamina to their different TCM clinical symptoms.^[20,21]^

Based on our years of clinical and experimental works in TCM, we suggest that through reinforcing spleen and kidney function and activating blood circulation, TCM could postpone CKD progress. According to that theory, we invented QGYS and it has been proven to be able to protect renal function and alleviate renal fibrosis in mouse experiments.^[22,23]^ We also conducted clinical researches on IgA-nephropathy patients and QGYS decoction achieved ideal therapeutic effects.^[24]^ This formula aims to treat Qi deficiency in spleen and kidney, which presents symptoms as sore waist and knee, hard breathing, spontaneous perspiration, pale face and hypodynamia, and so on. This decoction is of independent intellectual property rights and we are still searching its potential mechanisms of renal protection.

## 2 Methods/design

### 2.1 Study design

**Figure 1:**
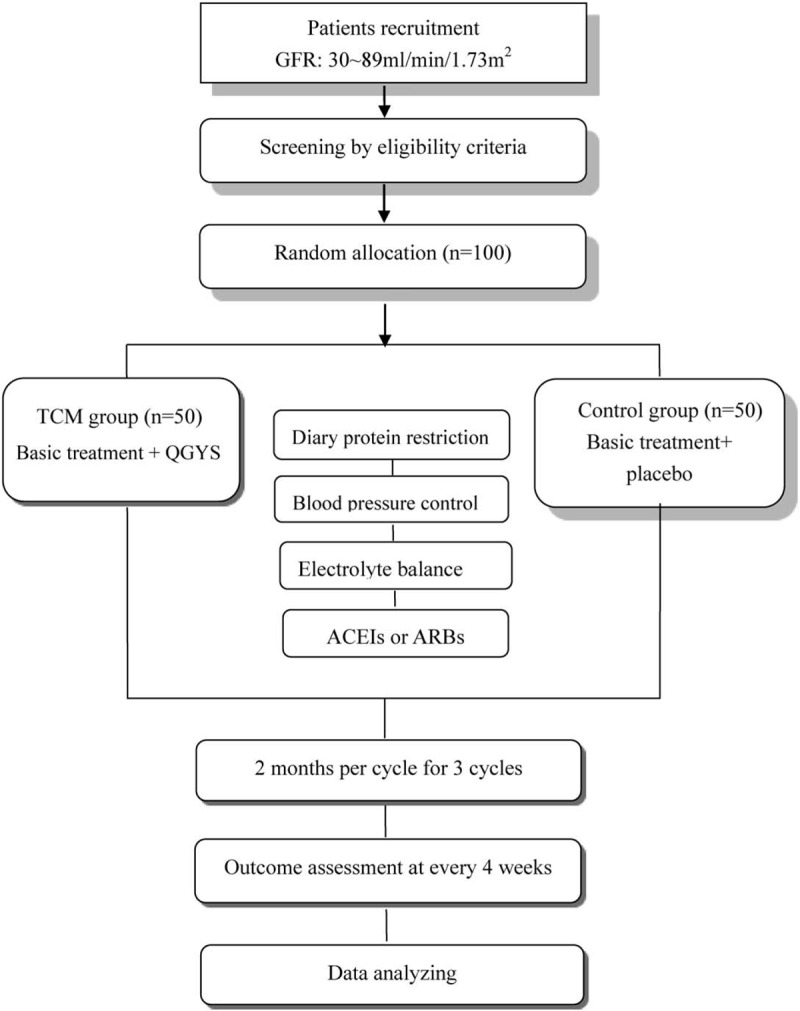
Flow diagram of this research.

A randomized, parallel, placebo controlled clinical trial will be conducted (Fig. 1). The protocol adheres to the Recommendations for Interventional Trials (SPIRIT) guidelines. This study aims to evaluate the efficacy and safety of Qi Gui Yi Shen decoction in treatment of patients with CKD. The study will follow the guidelines as illustrated in Figure 1. A total of 100 participants recruited form the First Affiliated Hospital of Soochow University in Suzhou are divided into TCM treatment group and placebo group in 1:1 ratio. Both groups are administrated with conventional western medicine treatment while TCM group receive extra Qi Gui Yi Shen decoction and placebo group receive placebo decoction. Patients take medicine 3 times a day for 2 months as 1 cycle. At the same time drug efficacy and safety will be evaluated. The total duration is 6 months in 3 cycles.

### 2.2 Objectives

The aim of this study is to evaluate the efficacy of conventional western medicine treatment combined with or without Qi Gui Yi Shen decoction in patients with CKD.

### 2.3 Study criteria

Patients diagnosed of CKD of stage II-III; TCM diagnose is spleen and kidney deficiency with syndromes as follows.

#### 2.3.1 Diagnostic criteria

Diagnostic criteria for CKD and stages: according to NKF-K/DOQI Clinical Practice Guideline by America National Kidney Foundation. Creatinine clearance is calculated according to CG GFR formulas: Ccr = (140−age) × weight × 1.23/Scr (if male) and Ccr = (140−age) × weight × 1.03/Scr (if female) for CKD stage determination.^[3]^

#### 2.3.2 TCM syndrome differentiation criteria

TCM syndrome differentiation is based on the Guidance Principles of Clinical Research on new Chinese medicine.^[4]^ Spleen and kidney deficiency syndromes include primary symptoms as weakness in waist and knees, shortness of breath and spontaneous perspiration, pale complexion, feeling spiritless; secondary symptoms including sallow complexion, heaviness of the head, foot edema, cold limbs, incontinence of urine, nocturia, and diarrhea (Table 1).

**Table 1:**
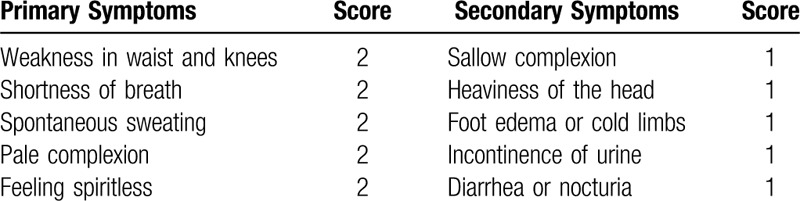
Spleen and kidney deficiency syndromes and the corresponding scoring.

#### 2.3.3 Inclusion criteria

- Patients with kidney function or structure injury for more than 3 months;
- Patients of stage II-III CKD: GFR:30∼89 ml/min/1.73 m^2^;
- Patients who have at least 2 main and 1 secondary symptoms of Spleen and kidney deficiency syndrome;
- Patients who are 18 to 75 years of age;
- Patients who agree to voluntarily participate in this study and sign informed consent.

#### 2.3.4 Exclusion criteria

- Patients who have been diagnosed with malignant cancer, sever heart, liver, brain or other organs dysfunction, shock, dehydration;
- Patients who are receiving emergent dialysis or kidney replacement treatment;
- Patients who are pregnant or planning to be pregnant;
- Patients with progressive neurological deficit or severe neurological symptoms;
- Patients who are judged to be inappropriate for the clinical study by the researchers.

### 2.4 Interventions

In addition to conventional western medicine treatment, TCM group will have additional Qi Gui Yi Shen decoction and control group have placebo.

#### 2.4.1 Basic treatment

According to Clinical Practice Guideline of CKD and dialysis from NKF-K/DOQI, all the patients are administrated with basic therapies as limiting intake of salt, protein and fat; managing electrolyte disturbance, anemia and coagulopathy as well as controlling blood pressure.

#### 2.4.2 Chinese herbal mixture

Apart from basic therapy, patients in TCM group are provided with QGYS decoction deprived from the following herbs: 30 g Radix Astragali, 15 g Radix Angelicae Sinensis, 15 g Radix Achyranthis Bidentatae, 15 g Rhizoma Chuanxiong, 15 g Radix Pseudostellariae, 15 g Rhizoma Atractylods Macrocephalae, 30 g Semen Eutyales, 30 g Fructus Rosae Laevigatae, 30 g Hedyotic Diffusa, 12 g Periostracum Cicadae and 6 g Radix Glycytthizae. Herbs are boiled in 600 to 700 ml water for 30 minutes and condensed into 300 ml decoction. Patients are administrated with 150 ml decoction three times a day after meal for 2 months.

#### 2.4.3 Placebo group

The placebo is made up by 10% of the Chinese herbs mixture, food color, and artificial flavors. Except for the dispensing technician, researchers and patients do not know the content of the drug packages.

### 2.5 Sample size

Sample size is determined for *t* test. To obtain a power of 90% (α = 0.05), and considering a drop-out rate of 20%, the total sample size required is determined to be 98, 49 in each group.

### 2.6 Randomization

Participants is randomly allocated to the controlled and TCM group with random number table. Numbers are generated and kept by a certain researcher who has no direct contact with the study participant. The randomized numbers will be kept in sealed envelopes, and random allocation will be conducted by opening an envelope as the researcher is informed of a participant's registration number. Before the randomization allocation, participants will be informed that they will be assigned to one of the 2 groups.

### 2.7 Blind

Because TCM syndrome differentiation is needed during the research process, in this trial only participants and the laboratory technicians as well as the biostatisticians responsible for the statistical analysis will be blinded to the assigned treatments.

### 2.8 Outcome measures

The primary outcome measure for this study is renal function and including estimated glomerular filtrate rate (eGFR), serum creatinine (Scr), blood urea nitrogen (BUN) and urinary protein creatinine ratio. They will be measured every 2 to 4 week.

The secondary outcomes include TCM symptoms changes (Table 2), cardiovascular function, lipid profile including triglyceride (TG), total cholesterol (TC), low density lipoprotein (LDL), high density lipoprotein (HDL), as well as serum levels of inflammatory mediators [i.e., TNF-α, IL-1β, IL-8, monocyte chemotactic protein-1 (MCP-1)].

**Table 2:**
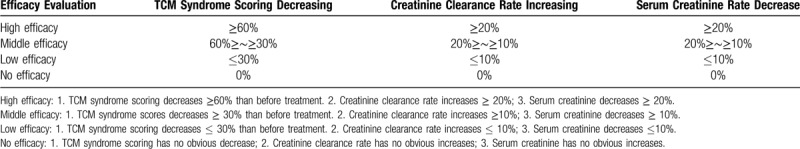
Evaluation of treatment efficacy by TCM syndrome scoring and Ccr and Scr variation.

**Figure 2:**
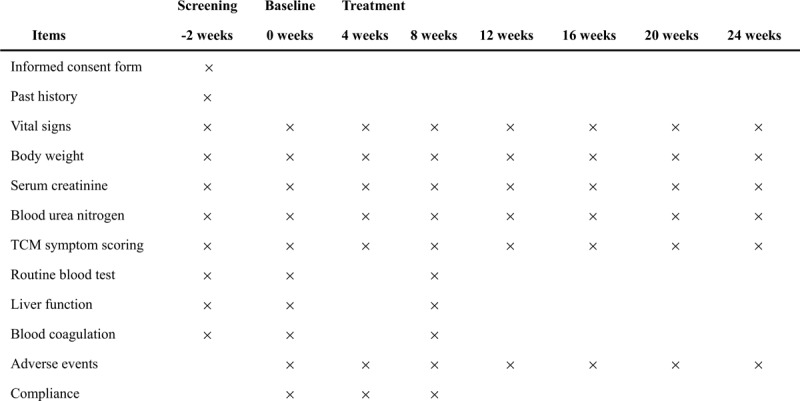
Schedule of enrollment, interventions and assessments.

Safety assessment: blood routine, liver function [i.e., alanine transaminase (ALT) and aspartate transaminase (AST)] and blood coagulation (Fig. 2).

### 2.9 Statistical analysis

Data analysis will be conducted with SPSS 15.0 for windows by professionals. The quantitative data are presented as mean ± standard deviation and analyzed by analysis of variance when normally distributed. Non-parametric data will be analyzed by Wilcoxon test. A paired *t* test will be used to analyze within the groups. A *P* value of less than .05 is considered as significant.

### 2.10 Data collection and monitoring

In this 6-month clinical trial, participants will take research medication for at least 8-week and 16-week follow-up. They need to pay regular visit to the research center and fill the evaluation questionnaire. The trial schedule is listed in Figure 2. Researchers are trained to collect trial data carefully according to standard protocol. Molecular biomarkers will be measured triplicated to assure quality. Quality control of data will be achieved throughout the trial process by the clinical center of the First Affiliated Hospital of Soochow University.

### 2.11 Ethical issues

This study is approved by the Ethics Committee of the First Affiliated Hospital of Soochow University and the trial protocol is registered at ChiCTR (www.chictr.org, trial identifier ChiCTR1900021622). Written informed consent will be obtained from each participant before enrollment. During the research, participants could withdraw from the trial for any reason at any time. Researchers could remove participants from the trial to ensure their safety or maintain the quality of the trial. Severe adverse event and unexpected adverse event will be reported to the Ethics Committee within 2 days.

## 3 Discussion

This protocol aims to evaluate the efficacy and safety of TCM Qi Gui Yi Shen decoction in the treatment of patients with CKD. CKD is now becoming increasingly common and hazardous while the current therapeutic options are limited, like angiotensin receptor blockers or angiotensin converting enzyme inhibitors usage, diary restriction and initiating diseases management. Their therapeutic effect is not satisfactory and the side effects are obvious. With the advances in clinical TCM theory and practice in kidney disease treatment, people tend to recognize TCM as an ideal complementary therapy and conduct extensive researches on the efficacy of TCM and the underlying mechanism, in the hope that better therapeutic effect could be achieved in more CKD patients. And this study will provide another new evidence of integrative therapy strategy for CKD.

## Author contributions

Yi Xi, Xun Lun, and Like Zhu are responsible for study design and drafting manuscript. Xiaoyi Sun, Yuqin Jiang and Minggang Wei obtain informed consent from potential trial participants. Weiming He supervises the experiment process, Xiaoyi Sun and Yuqin Jiang help to collect data. Weiming He and Minggang Wei contribute to the research design and made critical revisions. All authors have read and approved the final manuscript.
